# Work Stress Hampering Employee Performance During COVID-19: Is Safety Culture Needed?

**DOI:** 10.3389/fpsyg.2021.655839

**Published:** 2021-08-26

**Authors:** Farida Saleem, Muhammad Imran Malik, Saiqa Saddiqa Qureshi

**Affiliations:** ^1^Department of Management, College of Business Administration, Prince Sultan University, Riyadh, Saudi Arabia; ^2^Department of Management Science, COMSATS University Islamabad, Attock, Pakistan; ^3^Department of Business Administration, Fatima Jinnah Women University, Rawalpindi, Pakistan

**Keywords:** COVID-19, employee performance, work stress, safety culture, social exchange theory

## Abstract

During uncertain situations, such as the COVID-19 partial lockdown, maintaining satisfactory levels of employee performance (EP) is an important area of concern for many organizations. The current study examines the relationship of work stress due to COVID-19 (COVID-19 STR) and EP. Using social exchange theory (SET), safety culture (SC) is presented as a moderator for stress and performance relationships. A sample of 213 bank employees was collected using a convenient sampling method. Data were analyzed using stepwise linear regression and PROCESS Macro by Hayes (2013). Results revealed that COVID-19 STR has a negative impact on task and contextual performance (CP) and a positive impact on adaptive performance (AP). Similarly, the prevalence of SC significantly moderates the stress and performance relationships.

## Introduction

Stressful life situations such as pandemics can have significant negative implications for the mental health and psychological functioning of an individual. Stress, anxiety, mental confusion, social deprivation, and depression are a few examples of these mental and psychological issues (Yıldırım and Arslan, 2020). Brooks et al. (2020) also support that quarantined experiences due to COVID-19 lead to stress, fear, and frustration in individuals. Similarly, uncertainty due to COVID-19 is also associated with significant changes in our daily routines that can increase stress, depression, and anxiety (Arslan et al., 2020; Talaee et al., 2020; Mergel and Schützwohl, 2021). Likewise, a recent systematic review on the COVID-19 pandemic and mental health by Vindegaard and Benros (2020) and a narrative review on COVID-19 related mental health effects in the workplace by Giorgi et al. (2020) also concluded that COVID-19 has resulted in increased levels of depression, anxiety, and poor sleep quality. Other prior literature has investigated work stress having an impact on various work practices (Ram et al., 2011; Kinyita, 2015; Yunita and Saputra, 2019) under normal conditions, but the literature has not taken into account the effects of work stress on employee performance (EP) in uncertain conditions such as the outbreak of the COVID-19 pandemic.

Organizations continuously try to survive and sustain themselves (Bishwas and Sushil, 2016) requiring well-performing employees (Rani et al., 2013; Mensah et al., 2016). However, it becomes difficult for organizations to maintain consistency in their operations in uncertain external situations that can affect the well-being of their employees. These uncertain situations, such as the COVID-19 pandemic, can develop stress which hampers the performance of employees. The hazards prevailing in the work environment due to pandemics not only distract the attention of employees from work but also threaten their survival at the workplace by causing health problems (Carroll et al., 2009). In a recent study, Yunita and Saputra (2019) noted that a change in the environment is a common phenomenon that is frequently faced by employees in organizations. The changes taking place may cause stress among employees and this may lead to the generation of interpersonal conflicts that damage the working patterns of individuals (Kinyita, 2015). Correspondingly, stressed employees may experience depression and become unable to concentrate on their work, thus resulting in decreased performance (Yunita and Saputra, 2019).

The studies on workplace stress have considered two main streams that help in understanding how stress is created. The first stream highlights the traditional job-related stress (Roster and Ferrari, 2019; Yunita and Saputra, 2019; Jex and Beehr, 1991). These researchers examined how stressful psychosocial aspects of work environments, such as increased workloads, role conflict, lack of autonomy, and lack of social support, can lead to job strains and hamper performance (Beehr et al., 2001; Kinyita, 2015; Kożusznik et al., 2018). The second stream looked at the environmental aspects examining how worker abilities and their physical environment affects performance and how a person-environment “misfit,” if any, leads to adverse psychological or physiological responses (Lazarus and Cohen, 1977; Ram et al., 2011; Yunita and Saputra, 2019). However, there is another important perspective that can create stress in employees: uncertainty and threatful situations in the external environment. As such, elements of the physical environment interfere with workers’ ability to perform or pose undue demands on workers, thereby impeding the performance of employees by producing stress (McCoy and Evans, 2005).

The question now is how organizations can reduce the negative impact of COVID-19 stress on performance of employees. To address this, the current study synthesizes the literature of stress and performance with safety culture (SC). Social exchange theory (SET) is used for introducing SC as a moderator for COVID-19 stress and performance relationship. According to SET, social exchanges taking place in the organization fosters trust in employees. The provision of SC by the top management encourages employees to return by showing their sincere efforts toward achieving organizational goals (Cropanzano and Mitchell, 2005). SC can thus act as a boundary-condition between the relationship of stress and performance under situations created by COVID-19.

This study contributes to the existing literature in many ways; firstly, the current investigation is focused on the impact of external factors like the outbreak of COVID-19 on stress and performance relationship. However, internal organizational factors affecting employee stress and performance has dominated the existing literature. Secondly, the current study has developed and tested the proposed model in the banking sector of a developing country where, due to limited use of online banking services, the banking staff had to come to the bank during the outbreak of COVID-19. As the service providers, the employees working in the banks have more vulnerability toward prevailing hazards of COVID-19 due to the frequent human-to-human interactions. Similarly, we do find literature focusing on the stress and mental health of healthcare workers and its impact on their burnout (Luceño-Moreno et al., 2020; Yıldırım and Solmaz, 2020), work life balance (Magnavita et al., 2020), psychological wellbeing and anxiety (Denning et al., 2021; Galbraith et al., 2021; Mo et al., 2021), and their willingness to work (Maraqa et al., 2020) during the COVID-19 pandemic. However, very little is known about the impact of stress due to COVID-19 (COVID-19 STR) on the performance of banking staff who had to be available on duty during these tough times. Lastly, this study examines the influence of SC as a boundary-condition for the relationship between work COVID-19 STR and EP.

## Theoretical Framework

### Social Exchange Theory

The SET by Cook et al. (2013) gives a common understanding for how workers are likely to react when their psychological states are changed due to work pressures coming from the environment in which they work. According to SET, there exists a reciprocal relationship between the employees and their work environment. The SET assumes that all human relations are established on the basis of “cost-benefit” analysis and a comparability of alternatives (Nguyen et al., 2016). Previous studies recommend that psychological contracts help to explain the conditions of the social exchange relationship between employees and employers (Ahmad et al., 2019; Turnley et al., 2003). These relations comprise deliberate activities that all parties take part in with the assumption that all stakeholders will reciprocate these activities in one way or another.

The current study proposes a model based on the imminent work stress due to the COVID-19 situation and its impact on EP. The employees may feel more conscious about their health while coming to their workplaces, which may harm their work performance. There is an element of reciprocity where the managers who are willing to support their employees by ensuring their safety through safe working conditions are likely to receive reciprocity from employees in the form of higher performance levels. It is likely that SC will initiate social exchanges between the employees and their employers and will develop into a win-win situation for both parties.

**FIGURE 1:**
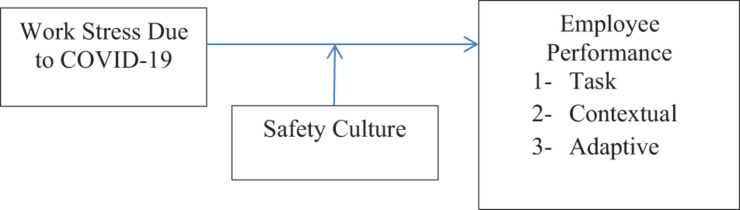
Proposed research framework.

The SET provides the foundation for the proposed research framework in two ways; first, the exchange is perceived by the employees in the presence of a threatening environment prevailing due to the COVID-19 pandemic. Secondly, the bank employees have to deal with various documents received via mail or in person from the customers coming to the bank. This exchange causes a threat to the employees and their morale may go down and may hamper their performance. Thirdly, the exchange takes place between the management and employees in the case of sharing SC and using ways for protection. The employees with a perception of safety being provided by the employer may remain committed to their work and hence show better performance outcomes, not only related to their tasks, but also toward maintaining good relationships in the workplace and adopting various ways to protect themselves and others by showing consistency in their work. Based on SET we propose a research framework in Figure 1.

## Literature Review

### Work Stress and Employee Performance

The EP is performing the job-related responsibilities diligently (Bartol, 1999; Briscoe and Claus, 2008). According to Atatsi et al. (2019), it is the degree to which employees fulfill the demands of the job assigned in a well-managed way within the given resources. EP is a combination of task performance (TP), contextual performance (CP), and adaptive performance (AP) (Koopmans et al., 2011). TP is related to performing the essential job-related tasks. Motowidlo (2000) views TP as involvement in accomplishing assigned tasks by an organization. Furthermore, it pertains to a person’s participation in attaining organizational efficiency by performing activities that are part of the official compensation system, and that details the specifications as highlighted in the job descriptions (Kuranchie-Mensah and Amponsah-Tawiah, 2016). Various terms represent the same concept, such as technical proficiency or in-role performance (Koopmans et al., 2013). TP is related to the employee actions that are formally prescribed in the job description and are evaluated by the management. Johnson and O’Leary-Kelly (2003) indicated that an employee’s level of TP is determined by their psychological ability rather than their sociability. Colquitt et al. (2012) indicated that psychological trust generates a sense of security in employees and makes them comfortable to keep task related exchanges with the organization.

Similarly, CP is viewed as behaviors that go beyond the officially described work objectives (Koopmans et al., 2011). It is a popular research subject studied in relation to companies and the person (Koopmans et al., 2013). Social facilitation and job commitment are the main characteristics of CP (Van Scotter, 2000). AP is about employees learning new things in their workplace. Individual task adaptability is the degree to which people deal with, react to, and/or support changes that affect their positions as a worker. It is affected by technological innovation, work enhancement, and changes in techniques, thus requiring workers to modify their workplace actions (Griffin et al., 2007). Adopting changes taking place in the workplace is about AP (Pulakos et al., 2002). Adaptive people are likely to have better concentration on their tasks, thus enhancing their TP (Shoss et al., 2012).

Among factors hampering employees’ performance, stress is the foremost factor that has negative implications (Ram et al., 2011; Kinyita, 2015; Yunita and Saputra, 2019). Stress is noted to have a negative effect on the psychological, behavioral, and physiological status of the individuals (Musyoka et al., 2012). It harms the motivation, morale, and performance of employees in the workplace. Moreover, it has a negative relationship with job satisfaction that interferes with one’s energy to work and results in lower performance levels (Ram et al., 2011). The stressed employees are faced with bad health conditions in addition to having worse work experiences in the workplace. This further decreases their energy to concentrate on their work and thus their performances decrease. The negative effects of work stress on the psychological and physical states of employees may threaten their health and result in damaged cognitive processes, short-term memory loss, and, in severe cases, hampered re-call of knowledge and distracted attention to the work assigned (Al-Hawajreh, 2011).

The sources of stress in the workplace, such as role overload, role ambiguity, and role conflicts, lead to disturbed flow of work. It is evident that the work stress and its more severe forms are increasingly prevalent in the work environment under the current circumstances of COVID-19 (Yıldırım and Solmaz, 2020; Denning et al., 2021; Galbraith et al., 2021; Mo et al., 2021). The employees who experience high degree of stress may have low commitment and satisfaction from their job (Saleem and Gopinath, 2015; Kuzey, 2018) that distracts their attention from their important work-related tasks and hampers their overall performance. Alternatively, the employees who feel more satisfaction at their workplace are more productive and have the capability to handle complex situations. The uncertainty due to the COVID-19 pandemic may lead toward disturbance in the flow of work and can result in role overload, role ambiguity, and role conflicts which may lead to increased stress and decreased performance in organizations. The banking sector is generally perceived as a stressful profession characterized by strict regulatory policies, heavy workloads, and ever-changing demands of the customers. These challenges can harm the psychological and physical health of the employees (Huber, 2000). Similarly, under the current COVID-19 situation, employees working in the banking sector are exposed to more stressful situations.

A combination of stressful events, such as the compliance pressures from the organization, interpersonal conflicts, and lack of professionalism, may affect the performance of employees. Inadequate skills to deal with the job and the mismatch between efforts made and the rewards received are common factors that create stress among employees and affect their TP (Bijleveld et al., 2011). Literature provides evidence for factors such as excessive workloads, inadequate workspace, inadequate resources, deficient company HRM policies, and strict deadlines (Botha and Pienaar, 2006) having negative effects on job performance. Similarly, the work stress hampers one’s self-efficacy and, as a result, employees feel they have less control over their work (Mo et al., 2021). This sense of lower self-efficacy may result in hampering CP by creating inadequacies in communication and damaged relationships with co-workers and managers. The stressed employees see their workplaces as having deficient social support and may develop a lack of trust (Wickham et al., 2014) that also negatively influences their CP. At the same time, the work stress may harm the thinking processes of employees and hinder their new practices’ adopting capacity. This slows down their pace of work, thus hampering their AP (Roster and Ferrari, 2019).

The perceptions of mistrust in any organization may lead to mental disturbances and employees to show compromising behaviors that negatively affect their AP (Saunders and Thornhill, 2004). Several studies have seen stress as having a negative effect on performance. However, it is not always the case. For example, Siswanto et al. (2019) noted work stress as a motivator to adopting new practices for better performance. In another recent study, Harras (2019) is argued that stress may invert the U-shaped relationship with employee’s performance. At first, the stress enhances motivation to work and after a certain threshold, it starts decreasing the motivation to work and diminishes performance due to unjustified work distribution, work irrelevance, complexity, and monotony. Moreover, it is argued that the performance of an individual improves with their capacity to handle work stress up to a certain threshold. However, after reaching that point, increased work stress may result in diminished performance (Jackson and Frame, 2018). Literature has also found a significant relationship between work stress and performance. Hence, based on the above literature findings, we propose that work COVID-19 STR in banking employees will have a significant impact on their tasks, both contextual and adaptive.

*H1a: Work stress due to COVID-19 significantly affects employees’ task performance*
*H1b: Work stress due to COVID-19 significantly affects employees’ contextual performance*
*H1c: Work stress due to COVID-19 significantly affects employees’ adaptive performance*

### Safety Culture as Moderator

A positive SC is characterized by communication based on mutual trust by shared perceptions of the importance of safety, and by confidence in the efficacy of preventive measures. The mere presence of safety standards or cursory implementation of these standards at the workplace cannot guarantee safety (Griffin and Neal, 2000). Proper implementation and application of these standards is required. It demands employees strictly follow safety standards to not only ensure their own safety but that of others as well. SC ensures the removal of errors and taking care of others that fosters trust among employees and supports performance by sharing their knowledge and skills, removing hazards, solving each other’s problems, and working without making mistakes, ensuring required standards of performance. The SC can be used to mitigate the negative effects of work stress on performance, especially when organizations are faced with the COVID-19 pandemic. It is further asserted that work stress is a function of internal and external forces, pressures, and cultures that require customized interventions (Muscroft and Hicks, 1998). Therefore, it is important to examine SC as an intervention to reduce the negative effects of work stress on EP relationships.

Ensuring safety in the workplace is very important for achieving performance targets as it ensures higher commitment and performance by employees. Several mechanisms can be developed to ensure EP through SC. Halligan and Zecevic (2011) view SC as “the product of individual and group values, attitudes, competencies, and patterns of behavior that determine the commitment to an organization’s health and safety programs.” The SC is the combined responsibility of all employees to ensure the commencement of safe working practices that foster trust and support among employees. It is noted that the availability of the high levels of support from supervisors and co-workers removes the negative effects of high-strain jobs and the levels of performance can be enhanced (Sargent and Terry, 2000), thus the working environment has a great influence on employees’ mental health.

Safety culture enables employees to learn the practices that are necessary to perform work from one another as illustrated by the SET (Griffin and Neal, 2000). The removal of errors helps employees become more engaged in their work and fosters extending support to one another while at the workplace. This leads them to minimize the stress levels as they perceive their workplace to be supportive and well managed, which enhances their capability of being productive. People who are satisfied with their work environment show extraordinary contributions toward their job (Hsu et al., 2016). SC protects employees against any harm in the organization. It provides the sense of being protected and makes employees satisfied while at their workplaces. This sense of safety keeps them motivated to perform well in the organization (Fulwiler and Gerlach, 2014). The employees who are safe and secure have better decision-making capabilities and they perform their work by using resources efficiently. The organizations with SC encourage developing positive work behaviors in their employees as they remain committed to safety and their work (Reason, 2000). While analyzing the impact of safety measures taken during COVID-19 on the work performance of Japanese employees, Sasaki et al. (2020) concluded that intensive implementation of workplace measures responding to COVID-19 reduce employees’ psychological distress and means they maintain their work performance.

In the light of the above findings, it is argued that the employees will be in a position to perform well when they feel safe at their workplaces. SC reduces potential threats prevailing in the work environment and provides a sense of satisfaction and reduced stress in employees (Didla et al., 2009). Moreover, it is noted that in an organizational context, the accidents are not the only danger that threaten the employees, develop stress, and negatively influence their performance; external environmental factors such as floods, earthquakes, and pandemics also have similar effects. Taking these different challenges on board, developing a SC seems paramount to providing a sense of protection to employees and helps in decreasing the stress due to uncertainty by making them feel safe, ensuring consistent performance. Due to the equivocal finding in literature regarding the impact of work stress on the performance of employees, we propose that under uncertain situations like the COVID-19 pandemic, SC acts as a boundary condition and moderator for relationships existing between work stress and performance. Hence, the following hypotheses are proposed:

*H2a: The safety culture moderates the relationship of work stress due to COVID-19 and employees’ task performance*
*H2b: The safety culture moderates the relationship of work stress due to COVID-19 and employees’ contextual performance*
*H2c: The safety culture moderates the relationship of work stress due to COVID-19 and employees’ adaptive performance*

## Methodology

### Sample and Data Collection

Bank employees were selected for this study because they were supposed to come to work even under the threatening situation of COVID-19. Along with their normal workload, the threatening conditions of COVID-19 added to their work stress, and they were deemed to be the most suitable people to respond to our questionnaire for examining the developed framework. The questionnaire used for data collection was presented to and approved by the ethical committee at COMSATS University Islamabad, Attock Campus, Pakistan. We have used a non-probability convenience sampling technique for data collection due to the COVID-19 situation as all bank employees were working from their offices. We planned a visit to different banks after contacting the branch manager via telephone and getting their approval for data collection from their branch employees. We visited sixteen different bank branches in Rawalpindi and Islamabad, Pakistan, collecting data in the month of May 2021. Bank employees willingly participated in the questionnaire survey. We contacted 375 bank employees working from their offices during the COVID-19 pandemic. We received 245 filled questionnaires. After careful scanning and due to missing information, we removed 32 responses. The response rate was 56% and the final usable sample size was 213. We selected the bank employees who were working from their offices during the COVID-19 situation and were working from 10am to 3pm (reduced working hours due to COVID-19). These employees were in direct or indirect contact with customers who visited the selected banks.

In the collected data, 53% of respondents were male and 47% were female. More than half of the respondents (69%) were between 36 and 45. Thirty-nine percentage respondents were working at the middle level of management in different branches of the banks followed by the first line managers (35%). It is also noted that nearly half of the respondents had 16 years of education (56%) and the majority of the respondents had work experience of 1–5 years (53%). The demographic information of respondents is presented in Table 1.

**TABLE 1:** Demographic information, *n* = 213.

**Variables**	**Category**	**Frequency**	**Percentage**
Gender	Male	112	53
	Female	101	47
Age (years)	26–35	20	9
	36–45	148	69
	46–55	45	21
Job status	Top level	55	25
	Middle level	83	39
	First level	75	35
Education	Below Graduation	16	15
	Graduation	62	29
	Masters	120	56
Experience (Years)	<1	30	14
	1–5	112	53
	6–10	49	23
	>10	22	10

*Source: field data.*

### Procedure

The bank employees were approached by seeking prior permission from the senior branch manager. All possible measures for social distancing (keeping at-least six feet distance) and personal protection (wearing masks and hand gloves and using sanitizer) were taken before handing over and retrieval of the questionnaires on both sides, researcher – respondent. Banks have already made these arrangements for the people visiting banks for their transactions and other purposes. The number of visitors was further controlled by making a queue outside the bank (each branch) and allowing a limited number of visitors at a given time. The questionnaire was written in plain English to avoid any confusion while reading the questionnaire. Anonymity and confidentiality were ensured, and the respondents were assured that their responses will not be shared with any manager or any other employee working in the bank. Moreover, it was ensured that the responses will only be used for the research purpose that gave employees more confidence to provide their genuine responses.

### Instrumentation

A closed-ended questionnaire was used to record the responses of bank employees who were working from offices during partial lockdown for COVID-19. All the statements for measuring the constructs were assessed on a five point Likert scale from “strongly disagree” as 1 to “strongly agree” as 5, except the demographic variables. We have collected data related to all variables at a single time due to difficulty in visiting the respondents again and again under the partial lockdown situation. The questionnaire was primarily compiled in English language which was easily completed by the bank employees as the official language of Pakistan is English and bank employees have enough educational background to understand the language.

#### Work Stress Due to COVID-19

Work COVID-19 STR was measured using the four-dimensions (anxiety, impact on duty, depressive symptoms, and sleep disturbance) scale adopted from Zaki et al. (2020). Each dimension was measured with separate questions. Anxiety due to COVID-19 was measured using nine questions. Sample questions are “I can’t stop imagining catching COVID-19” and “I feel helpless.” COVID-19 impact on duty was assessed with the help of three questions and the sample question is “has COVID-19 impacted your employment?” Depression symptoms were measured with the help of three questions. The sample question is “I lost motivation and interest in aspects of life.” Sleep disturbance was also measured with the help of three questions and the sample question is “My sleep/wake routine is different after COVID-19.”

#### Employees’ Performance

The questionnaire for the employees’ performance was adopted from Koopmans et al. (2013, 2014) with three underlying dimensions: TP, CP, and AP. TP was measured with the help of seven items adopted from Koopmans et al. (2014). The sample items are “In the past 3 months I managed to plan my work so that it was done on time” and “In the past 3 months I was able to perform my work well with minimal time and effort.” 8-itemed CP scale adopted from Koopmans et al. (2014) was used for the measurement of CP of respondents. The sample items are “In the past 3 months I took on extra responsibilities” and “In the past 3 months I came up with creative solutions to new problems.” The AP was measured with the help of a 5-itemed scale adopted from Koopmans et al. (2013). The sample item is “I was able to cope well under uncertain and unpredictable situations at work.”

#### Safety Culture

The scale for the SC was adopted from Lee et al. (2017) with five items. They have reported Cronbach’s alpha value of 0.884. The sample items included in the SC scale were “I feel safe by being here,” “I am encouraged by my colleagues to report any safety concerns I may have,” and “the culture in this organization makes it easy to learn from the errors of others.”

## Data Analysis and Results

### Control Variables

We have used ANOVA test to identify the significant impact of the demographic variables on the proposed model. It was identified that the experience of respondents was not significantly related to any variable presented in the model. Education was related to AP (*F*: 3.6; *p*: 0.05). Job status was significantly related to AP (*F*: 6.3; *p*: 0.01). Age was related to CP (*F*: 2.7; *p*: 0.07) and COVID-19 STR (F: 3.3; p: 0.04). Gender was significantly related to SC (*F*: 4.7; *p*: 0.03). Hence, all these variables were taken as control variables for further analysis.

### Scale Validation

For scale validation we have performed both exploratory and confirmatory factor analysis (CFA) for work COVID-19 STR and performance scale. We have used the exploratory factor analysis for these two constructs because they have their underlying dimensions. The parameter values of Kaiser-Meyer-Olkin and Bartlett’s sphericity test provide justification for the use of exploratory factor analysis technique for work COVID-19 STR and performance scales. We have used principal component analysis with varimax rotations as a factor extraction method. The exploratory factor analysis identified the underlying dimensions of COVID-19 STR and performance scales. After exploratory factor analysis we have conducted CFA using AMOS 17. The CFA, also called the measurement model, was run taking work COVID-19 STR as second order factor with underlying dimensions. The measurement model with latent and observed factors produces acceptable model fit indices. The results of CFA are presented in Table 2.

**TABLE 2:** Results of confirmatory factor analysis (CFA).

**Construct/Variable**	**Factor loadings**	**Alpha**	**CR**	**AVE**
**Stress due to COVID 19:**				
Anxiety and fear		0.92	0.92	0.58
ANX1	0.694			
ANX2	0.838			
ANX3	0.748			
ANX4	0.853			
ANX5	0.805			
ANX6	0.798			
ANX7	0.848			
ANX8	0.607			
ANX9	0.621			
Impact on duty		0.91	0.92	0.79
IOD1	0.899			
IOD2	0.893			
IOD3	0.875			
Depression		0.88	0.89	0.74
DEP1	0.794			
DEP2	0.945			
DEP3	0.832			
Sleep disturbance		0.79	0.79	0.56
SLD1	0.758			
SLD2	0.810			
SLD3	0.679			
Safety culture		0.90	0.90	0.65
SC1	0.753			
SC2	0.754			
SC3	0.795			
SC4	0.852			
SC5	0.880			
Task performance		0.96	0.96	0.80
TP1	0.883			
TP2	0.879			
TP3	0.939			
TP4	0.891			
TP5	0.879			
TP6	0.867			
TP7	0.910			
Contextual performance		0.97	0.97	0.82
CP1	0.916			
CP2	0.902			
CP3	0.893			
CP4	0.896			
CP5	0.881			
CP6	0.906			
CP7	0.901			
CP8	0.945			
Adaptive performance		0.92	0.92	0.70
AP1	0.756			
AP2	0.869			
AP3	0.865			
AP4	0.887			
AP5	0.803			

*Goodness of fit indices.*

*χ^2^ = 1642; d.f. = 830; χ^2^/d.f. = 1.98; *p* < 0.001; CFI = 0.91; GFI = 0.75; AGFI = 0.71; RMR = 0.05; RMSEA = 0.07.*

### Reliability and Validity

The output of the measurement model was used for reliability and validity measurement checks. For reliability, we have used Composite Reliability and Cronbach Alpha values. The values of both indices were greater than the proposed cutoff value of 0.70 (Nunnally and Bernstein, 1994). Similarly, for validity we have used AVE values that were more than 0.5, convergent validity where all observed variables were successfully loaded into their respective construct. Results are presented in Table 2

Lastly, we checked the discriminant validity using Fornell and Larcker (1981) criterion, where the values of AVE for all constructs were greater than the shared variances. Results of correlation and discriminant validity are presented in Table 3.

**TABLE 3:** Descriptive statistics and correlations.

	**Variable**	**No of items**	**Mean**	**s.d.**	**1**	**2**	**3**	**4**	**5**
1	COVID-19 STR	18	3.83	0.63	**0.67**				
2	SC	5	2.14	0.79	−0.02 (0.30)	**0.65**			
3	TP	7	2.26	1.07	−0.15** (0.27)	0.33* (0.24)	**0.80**		
4	CP	8	2.40	1.10	−0.12*** (0.24)	0.28* (0.30)	0.83* (0.36)	**0.82**	
5	AP	5	3.19	0.69	0.18* (0.18)	0.20* (0.21)	0.31* (0.34)	0.21* (0.32)	**0.70**

*COVID-19 STR, stress due to COVID-19; SC, safety culture; TP, task performance; CP, contextual performance; AP, adaptive performance.*

Shared variance in parenthesis; AVE in diagonal; *P < 0.01; **P < 0.05; ***P < 0.10; s.d., standard deviation; AVE in bold and diagonal.

### Hypotheses Testing

#### Stepwise Linear Regression Analysis

To test the first set of hypotheses (H1a, H1b, and H1c) we have used stepwise linear regression in SPSS. In the first step we entered all control variables and in the next step we entered the independent variable and the moderator. The results of three stepwise linear regression analysis with respect to each dependent variable are presented in Table 4.

**TABLE 4:** Stepwise linear regression.

	**DV: TP**		**DV: CP**		**DV: AP**	
	**Un-standardized coefficient**	***t*-Value**	**Un-standardized coefficient**	***t*-Value**	**Un-standardized coefficient**	***t*-Value**
**Step1 (Control variables)**						
Gender	0.016	0.239	0.138	2.083**	0.049	0.731
Age	0.039	0.592	0.010	0.146	−0.076	−1.121
Education	0.077	1.204	0.058	0.887	0.064	0.968
Experience	−0.040	−0.620	−0.017	−0.253	0.012	0.178
Job Status	−0.065	−0.995	−0.013	−0.197	−0.104*	−1.736*
**Step2 (Independent variables)**						
COVID-19 STR	−0.258*	−2.369*	−0.225**	−2.002**	0.173*	2.360*
SC	0.447*	5.167*	0.385*	4.221*	0.166**	2.873*
**Model fit**						
*F*-value	15.91		10.96	6.28		
R2	0.13		0.10	0.10		
*p*-value	0.00		0.00	0.00		

COVID-19 STR, stress due to COVID-19; SC, safety culture; TP, task performance; CP, contextual performance; AP, adaptive performance; *p < 0.01; **p < 0.05; ***p < 0.10.

The results identified that COVID-19 STR has a significant negative impact on TP (*B* = −0.258; *P* < 0.01) and CP (*B* = −0.225; *P* < 0.05) while it has a significant positive impact on AP (*B* = 0.173; *P* < 0.01) in the presence of control variables and moderator. Hence, H1a, H1b, and H1c are accepted.

#### Moderation Analysis

For the testing of the second set of moderation hypotheses (H2a, H2b, and H2c), we conducted a moderated regression analysis by using PROCESS Macro (extension in SPSS) by Hayes (2013). We preferred to use PROCESS Macro over SEM and simple regression analysis due to its robustness. PROCESS Macro uses a bootstrapping approach with biased corrected 95% confidence intervals and calculates the Johnson-Neyman outputs for the interaction term. We have used Model No 1 of PROCESS Macro by centering the variables that define product term and conditioning values at mean and ± 1SD. Johnson-Neyman outputs for the interaction term were also calculated. Results are presented in Table 5.

**TABLE 5:** Five thousand bootstrap results for process model no.1 simple moderation analysis.

	**DV: TP**				**DV: CP**				**DV: AP**			
	**Estimate**	**SE**	**LL 95% CI**	**UL 95% CI**	**Estimate**	**SE**	**LL 95% CI**	**UL 95% CI**	**Estimate**	**SE**	**LL 95% CI**	**UL 95% CI**
Gender	0.092	0.143	−0.189	0.373	0.354**	0.149	0.061	0.649	0.071	0.095	−0.116	0.257
Age	0.076	0.134	−0.189	0.341	0.062	0.141	−0.215	0.339	−0.104	0.089	−0.280	0.071
Education	0.093	0.095	−0.084	0.294	0.087	0.100	−0.110	0.285	0.038	0.064	−0.087	0.163
Experience	−0.031	0.083	−0.195	0.133	0.037	0.087	−0.135	0.285	0.008	0.055	−0.101	0.117
Job Status	−0.094	0.091	−0.273	0.085	−0.043	0.095	−0.230	0.144	−0.105*	0.060	−0.223	0.014
COVID-19 STR	−0.271*	0.113	−0.047	−0.495	−0.269**	0.119	−0.034	−0.504	0.220*	0.075	0.072	0.369
SC	0.444*	0.087	0.271	0.617	0.406*	0.092	0.225	0.587	0.164*	0.058	0.279	0.049
COVID-19 STR* SC	0.362**	0.172	0.022	0.702	0.377**	0.180	0.021	0.732	0.273**	0.114	0.047	0.498
**Model Fit**												
*F*-value	5.03*				4.05*				3.40*			
R2	0.16				0.14				0.12			
R2 Change	0.02**				0.18**				0.03**			

COVID-19 STR, stress due to COVID-19; SC, safety culture; TP, task performance; CP, contextual performance; AP, adaptive performance; *p < 0.01; **p < 0.05; ***p < 0.10.

**FIGURE 2:**
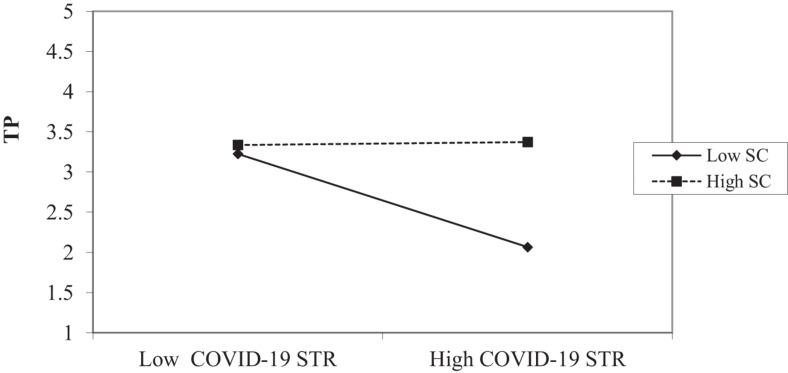
Interaction effects of stress due to COVID-19 (COVID-19 STR) and safety culture (SC) on task performance (TP).

The results identified that all interaction terms were significant and there is no zero in the lower and upper bound of 95% confidence interval of interaction terms. We plotted an interaction graph for low and high (Mean ± SD) values of moderator (SC) for all proposed moderation hypotheses. The interaction graph of COVID-19 STR and TP relationship (shown in Figure 2) suggests that this relationship is significant for SC. The slope test shows that the slope for low SC is insignificant (*B* = −0.018, ns) while it is significant for high SC (*B* = 0.559, *P* < 0.01). This finding supports H2a, which suggests that in case of high COVID-19 STR, individuals who perceive high SC show high TP whereas in cases of high COVID-19 STR, individuals who perceive low SC show low TP.

**FIGURE 3:**
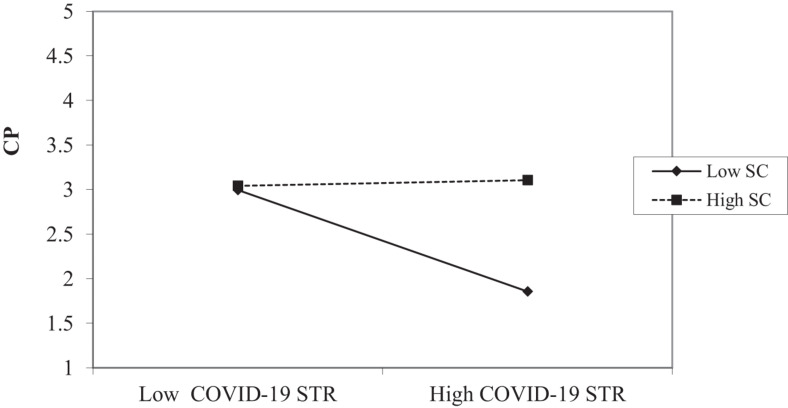
Interaction effects of COVID-19 STR and SC on contextual performance (CP).

The interaction graph of COVID-19 STR and CP relationship (shown in Figure 3) suggests that this relationship is significant for SC. The slope test shows that the slope for low SC is insignificant (*B* = −0.031, ns) while it is significant for high SC (*B* = 0.569, *P* < 0.01). This finding supports H2b, which suggests that in cases of high COVID-19 STR, individuals who perceive high SC show high CP whereas in cases of high COVID-19 STR, individuals who perceive low SC show low CP.

**FIGURE 4:**
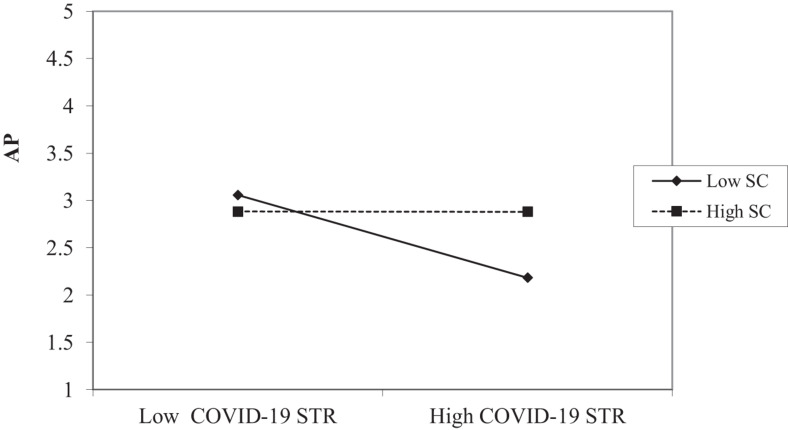
Interaction effects of COVID-19 STR and SC on adaptive performance (AP).

The interaction graph of COVID-19 STR and AP relationship (shown in Figure 4) suggests that this relationship is significant for SC. The slope test shows that the slope for low SC is insignificant (*B* = 0.003, ns) while it is significant for high SC (*B* = 0.438, *P* < 0.001). This finding supports H2c, which suggests that in cases of high COVID-19 STR, individuals who perceive high SC show high AP whereas in cases of high COVID-19 STR, individuals who perceive low SC show low AP.

## Discussion

Through this study we have examined the relationship of work COVID-19 STR and the performance of banking sector employees during partial lockdown. By using SET, we also propose the moderating role of SC for the relationships of work COVID-19 STR and EP. We have treated EP as a three-dimensional construct having task, contextual, and adaptive dimensions. The first important finding is related to the relationship of COVID-19 stress and task and CP. The results revealed that work COVID-19 STR has a significant negative impact on task and CP of banking sector employees. However, this negative impact is much stronger for TP followed by CP. These results are consistent with the prior literature on work stress and EP in general (Jex and Beehr, 1991; De Ruyter et al., 2001; Saleem and Gopinath, 2015; Kożusznik et al., 2018; Roster and Ferrari, 2019) and under the COVID-19 pandemic in particular (Giorgi et al., 2020; Garcia et al., 2021; Kumar et al., 2021).

The employees experiencing work stress feel that their autonomy is decreasing, and they may not perform as per the required standards, thus resulting in low TP (Pulfrey et al., 2013). At the same time, the employees working in a risky environment also look for increased pay packages and if they are not given the increased packages or the allowance to work in a threatened environment, that also contributes toward their decreased productivity. The health hazards not only affect the individuals in their individual capacity but also add to the cost of the organization in terms of employee absence and turnover of employees (McCalister et al., 2006).

The second important finding of the current investigation is related to the relationship of COVID-19 STR and AP. The results found that work COVID-19 STR has a significant positive impact on AP. This result is also consistent with some recent findings. Garcia et al. (2021), after studying the impact of COVID-related stress in university faculty members, found both positive and negative effects of stress on work performance. Wong et al. (2021) also supported the positive impact of COVID-19 related stress on the job performance while examining the responses of hotel employees in the United States. Similarly, Siswanto et al. (2019) identified work stress as a motivator to adopt new practices that may be useful for protecting employees from the harmful effects of COVID-19 while performing at work. Our finding related to AP is in line with the notion that stressors can act as motivators for determining enhanced job performance (McGowan et al., 2006). According to this notion, under stressful situations people try their best to use newer ways to perform their work and to perform better and quicker than others in the workplace. Work stress motivates employees for adoption of better ways to work, thus enhancing their AP in the workplace. The characteristic of having adaptability encourages employees to learn more and use their knowledge in a direction to achieve organizational goals.

Similarly, this result also supports that employees want to adopt practices that make them safe from COVID-19. The flexibility in working allows them to adopt new ways of doing work through the use of information technology, internet, and other communication technologies. This adaptability helps them perform well while doing their office work without getting stressed out due to COVID-19. The adaptive people quickly understand the requirements of the work environment and respond quickly without compromising their work routines (Shoss et al., 2012) as the performance of an individual is significantly affected by their capacity to handle work stress (Yunita and Saputra, 2019).

The third important finding of this study is related to the proposed moderating role of SC. Results provide support for the SC as a significant moderator. Our finding related to SC is consistent with the finding of Sasaki et al. (2020) who found that rigorous application of workplace measures responding to COVID-19 reduce employees’ psychological distress and maintains their work performance. With this finding, we generalize the SET in the COVID-19 context. According to this theory, social exchanges that are taking place between the top managers and the staff help in strengthening SC in the organizations. The safety measures taken by an organization trigger reciprocity behavior in the form of high performance by employees. Due to safety measures taken by organizations and maintaining the SC, employees feel safe and secure, which not only reduces their stress levels but also positively impacts their performance. Moreover, the exchange takes place between the management and employees in the form of sharing SC and using ways for protection from situations such as COVID-19 help in the maintenance of SC in the organization. Employees with perceptions of safety may remain committed to their work and hence show better performance. Due to COVID-19, it appears that people are more concerned about themselves and others and with helping them, in addition to ensuring the safety measures that contribute positively toward their performance at their workplaces.

## Practical Implications

Our study has several important practical implications. One important implication for managers in the banking sector is to anticipate the possible adverse effects of COVID-19 STR on EP. Managers need to understand that in the fast-paced banking industry, the employees are already working in stressful situations. The fear and COVID-19 STR can further increase the stress levels of employees. The COVID-19 outbreak is significantly impacting employees’ work and non-work lives that is resulting in the development of anxiety, frustration, and burnout, further leading to health problems that affect their work performance. If the managers are unable to take care of the anxiety and COVID-19 STR, it may lead to employees’ decreased engagement, poor work quality, and errors, eventually threatening the organization’s survival in these difficult times. The strategies that can possibly help managers to cope with these difficult times include developing a sense of a safe and secure work environment and full-time availability of support from the organization. Although stress has a negative impact on certain aspects of an employee’s performance, the intervention of SC may prove to be a stress management tool that helps in decreasing stress and improving performance.

Another practical implication is taking SC as a base for enhanced performance. The managers can use the technology to keep distance between employees and between employees and customers so that they feel safe and work with diligence. Furthermore, the “at-home” work structures are a useful way to continue work as well without being threatened by the COVID-19 spread. The managers must focus on the employee’s AP and develop mechanisms to reward it in an effective way. The COVID-19 has become an accelerator for workplace transformations. The employee’s AP has gained more importance in the times of COVID-19. Hence, the individuals and organizations who adopt the precautionary measures quickly will face less stress and uninterrupted performance.

Lastly, training is seen to have positive effects on the performance of employees but, in the times of COVID-19, it becomes inevitable to train employees to safeguard themselves from the threats of COVID-19 for better performance outcomes (Giorgi et al., 2020). Additionally, the frequent communication from the top managers regarding protection measures and the facilities available at the bank will help employees to have good performance.

## Limitations and Future Directions

The major limitation of our study is that data were collected through self-reported measures and cross-sectional sampling design which might produce common method variance (Podsakoff et al., 2003). We have used Herman’s single factor analysis to rule out common method variance issues. However, future studies can use longitudinal sampling design and collect data from supervisors and subordinates to limit common method variance. The second limitation is related to the generalizability of results to sectors other than banking and to developed economies as the infrastructure and availability of technology is quite variable in developed and developing economies. For generalizability of results of the current study, future investigations can collect data from other sectors and developed countries. Lastly, we have considered only one boundary condition “SC”; future studies can include other moderators and/or explanatory variables as an extension of the current model for better understanding of how stress is linked with positive and negative effects on performance.

## Conclusion

To meet the challenges of banking during COVID-19, the job performance of employees is of vital importance. Decreasing stress by maintaining a SC is necessary for improving the TP of employees. At the same time, the existence of a certain level of stress boosts the AP of employees. SET seems effective in terms of developing exchange relationships within an organization, shaping the SC and strengthening the task, contextual, and AP. Furthermore, it is concluded that not only is TP important in banks, but behavioral performance (CP) and using new ways to get to the targets (AP) are also important.

## Data Availability Statement

The raw data supporting the conclusions of this article will be made available by the authors, without undue reservation.

## Author Contributions

FS has developed the overall manuscript and collected data. MM has done the proofreading and data analysis for the manuscript developed. SQ has assisted in data in revised data collection and revisions of this manuscript. All authors contributed to the article and approved the submitted version.

## Conflict of Interest

The authors declare that the research was conducted in the absence of any commercial or financial relationships that could be construed as a potential conflict of interest.

## Publisher’s Note

All claims expressed in this article are solely those of the authors and do not necessarily represent those of their affiliated organizations, or those of the publisher, the editors and the reviewers. Any product that may be evaluated in this article, or claim that may be made by its manufacturer, is not guaranteed or endorsed by the publisher.
